# Supplementary figures and images for: Modulation of flagellar rotation in surface-attached bacteria: A pathway for rapid surface-sensing after flagellar attachment
Source: PLoS Pathog. 2019 Nov 4;15(11):e1008149. doi: 10.1371/journal.ppat.1008149 (PMC6855561; doi:10.1371/journal.ppat.1008149)

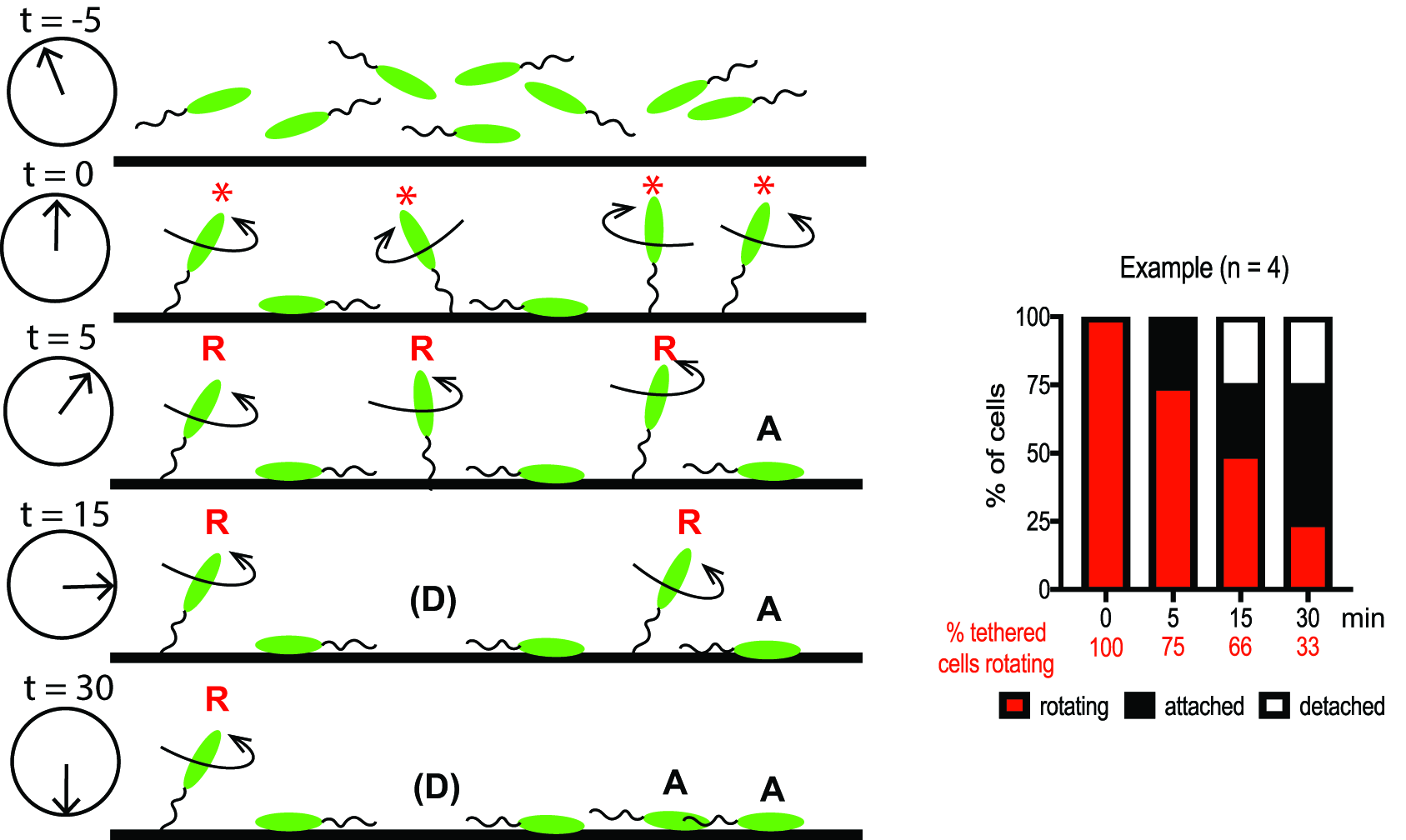

Supplement: S1 Fig — Bacteria were incubated with an anti-FliC antibody-coated slide for 5 minutes prior to initial imaging. After 5 minutes (t = 0), bacteria were scored as either attached/spinning or attached/not spinning; in this example, we scored 66% spinning, 33% not spinning. All bacteria identified as spinning were then followed by obtaining 1 minute movies at the indicated time points. Every cell scored as spinning at t = 0 was categorized as still rotating (“R”), no longer rotating and horizontally attached to the surface (“A”), or detached from the slide (“D”) based on analysis of these movies. The bar graphs show the fate of each cell scored as spinning at t = 0; by definition, 100% of tethered cells are rotating at t = 0. The “% tethered cells rotating” indicates the percentage of rotating cells/ (rotating cells + attached cells). (TIF) [file ppat.1008149.s001.tif]

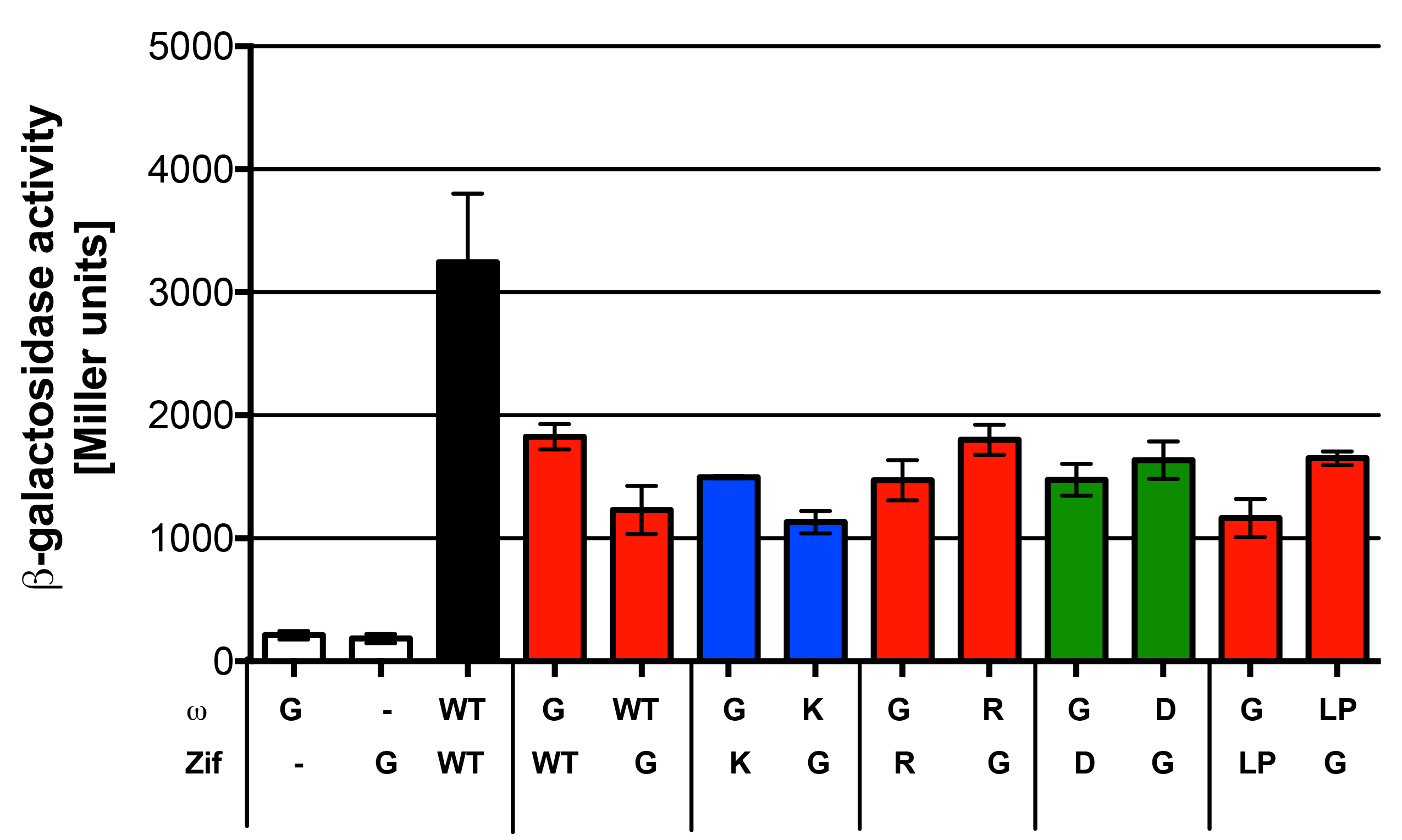

Supplement: S2 Fig — ω or Zif fusions to FliG and to wild-type and mutant alleles of FlhF were constructed as indicated, with interactions resulting in beta-galactosidase expression and activity (reported in Miller units). Bars show mean ± S.D. (n = 3) for a representative experiment. The FlhF homodimer (“WT”), serves as a positive control (black bar). FliG (“G”) interacted with all tested alleles of FlhF, including the hydrolytically active wild-type (“WT”) and FlhF(L298R, P299L) (“LP”) alleles, the GDP-locked FlhF(R251G) (“R”), as well as alleles defective in GTP hydrolysis (FlhF(K222A), “K”) or binding (FlhF(D294A), “D”). No signal was observed when FliG was co-expressed with either the ω or Zif domain alone (white bars). (TIF) [file ppat.1008149.s002.tif]

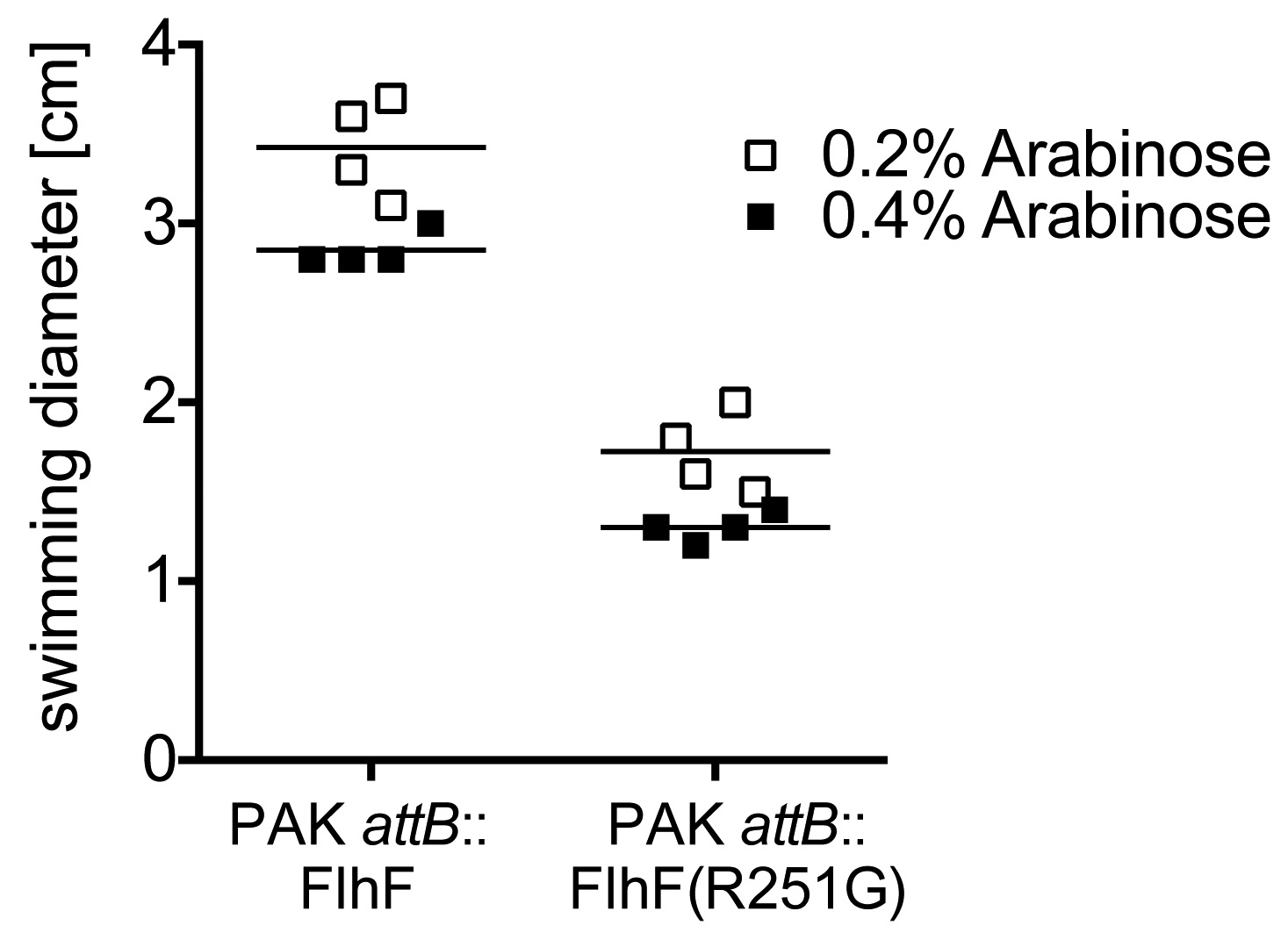

Supplement: S3 Fig — A second copy of flhF or flhF(R251G) was integrated into the attB site of PAK and expressed from an inducible arabinose promoter. Swimming zone diameter was determined in the presence of 0.2% (open symbols) and 0.4% arabinose (solid symbols); lines indicate means for each condition. Over-expression of FlhF(R251G) significantly inhibited swimming motility (***, p < 0.001; 2way ANOVA with Bonferroni post-test). (TIF) [file ppat.1008149.s003.tif]

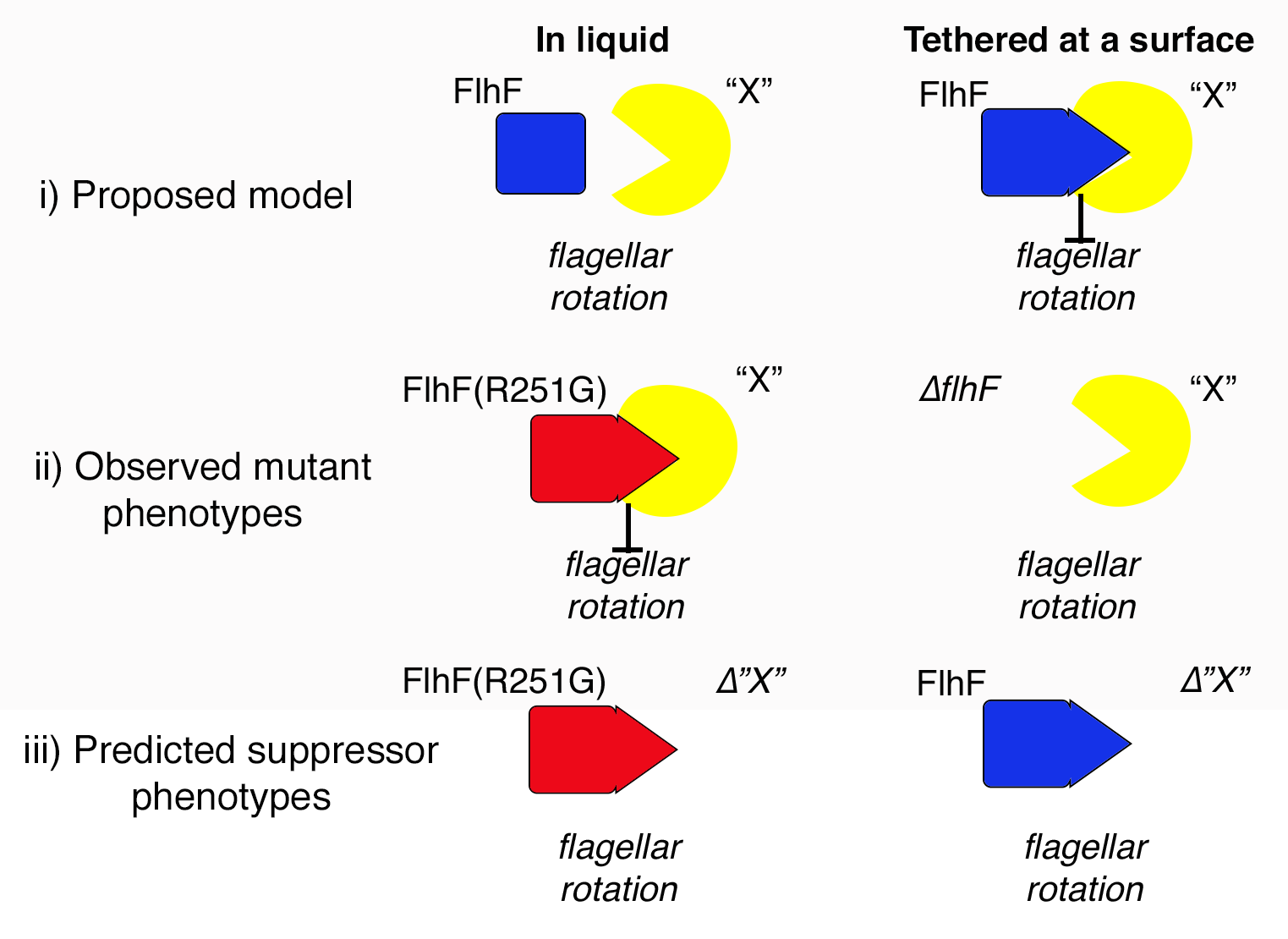

Supplement: S4 Fig — We propose that FlhF interacts with an unknown protein (“X”) to stop flagellar rotation when bacteria attach to a surface via their flagellum. In the case of a FlhF(R251G) mutation, the mutant FlhF(R251G) protein adopts a conformation that allows it to interact with protein X even when bacteria are not tethered at a surface. We also observe that ΔflhF bacteria fail to stop flagellar rotation when tethered at a surface and hypothesize that this results from an absence of the FlhF-Protein X interaction. We predict that we can identify suppressors that disrupt the FlhF-Protein X interaction by finding bacteria that can swim in liquid despite expression of FlhF(R251G). Our model also predicts that if these suppressor mutations are introduced into the wild-type background, they will phenocopy a ΔflhF mutant and fail to stop flagellar rotation after bacterial tethering to a surface. (TIF) [file ppat.1008149.s004.tif]

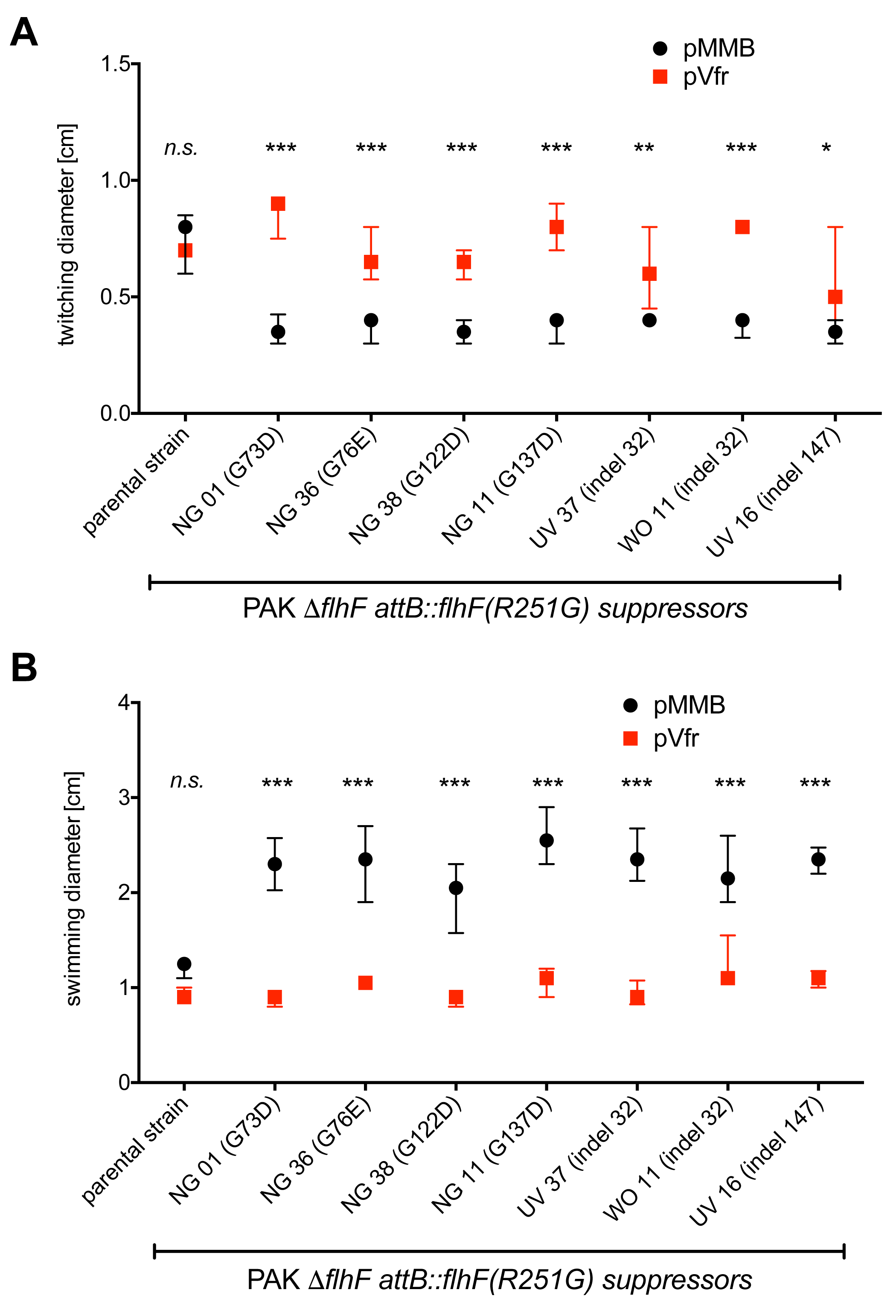

Supplement: S5 Fig — Suppressor mutants mapped to vfr were transformed with plasmid-encoded wild-type Vfr (red) or empty vector (black) and assayed for motility. Missense mutations and the amino acid position preceding indels are indicated for each suppressor. (A) Twitching motility of suppressors is complemented in trans by wild-type Vfr. Each symbol represents the median of 6–10 technical replicates; the error bar shows the interquartile range. Complementation with wild type Vfr had a significant effect on twitching motility of all suppressor mutants, but not on the parental strain PAK ΔflhF + attB::flhF(R251G) (ns, p > 0.05). (B) Complementation of vfr suppressors in trans with wild-type Vfr reverts cells to a paralyzed swimming phenotype. Each symbol shows median ± interquartile range of ≥ 10 technical replicates. Over-expression of wild type Vfr had a significant effect on all suppressor strains, but not on the parental strain. (Two-way ANOVA with Bonferroni post-test; *, p < 0.05; **, p < 0.01; ***, p < 0.001; n.s., p > 0.05.). (TIF) [file ppat.1008149.s005.tif]

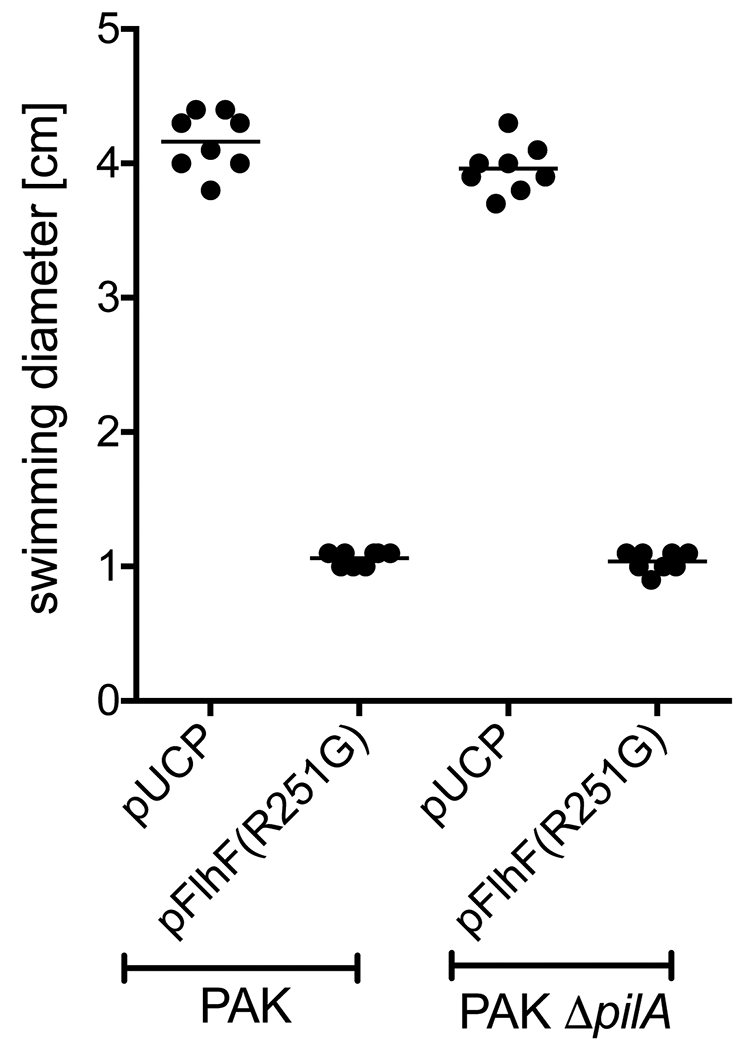

Supplement: S6 Fig — FlhF(R251G) was over-expressed in wild-type PAK and the isogenic pilA mutant. In both strains the dominant negative effect of FlhF(R251G) on swimming was observed. Each point represents a technical replicate swimming assay; lines indicate means. (TIF) [file ppat.1008149.s006.tif]

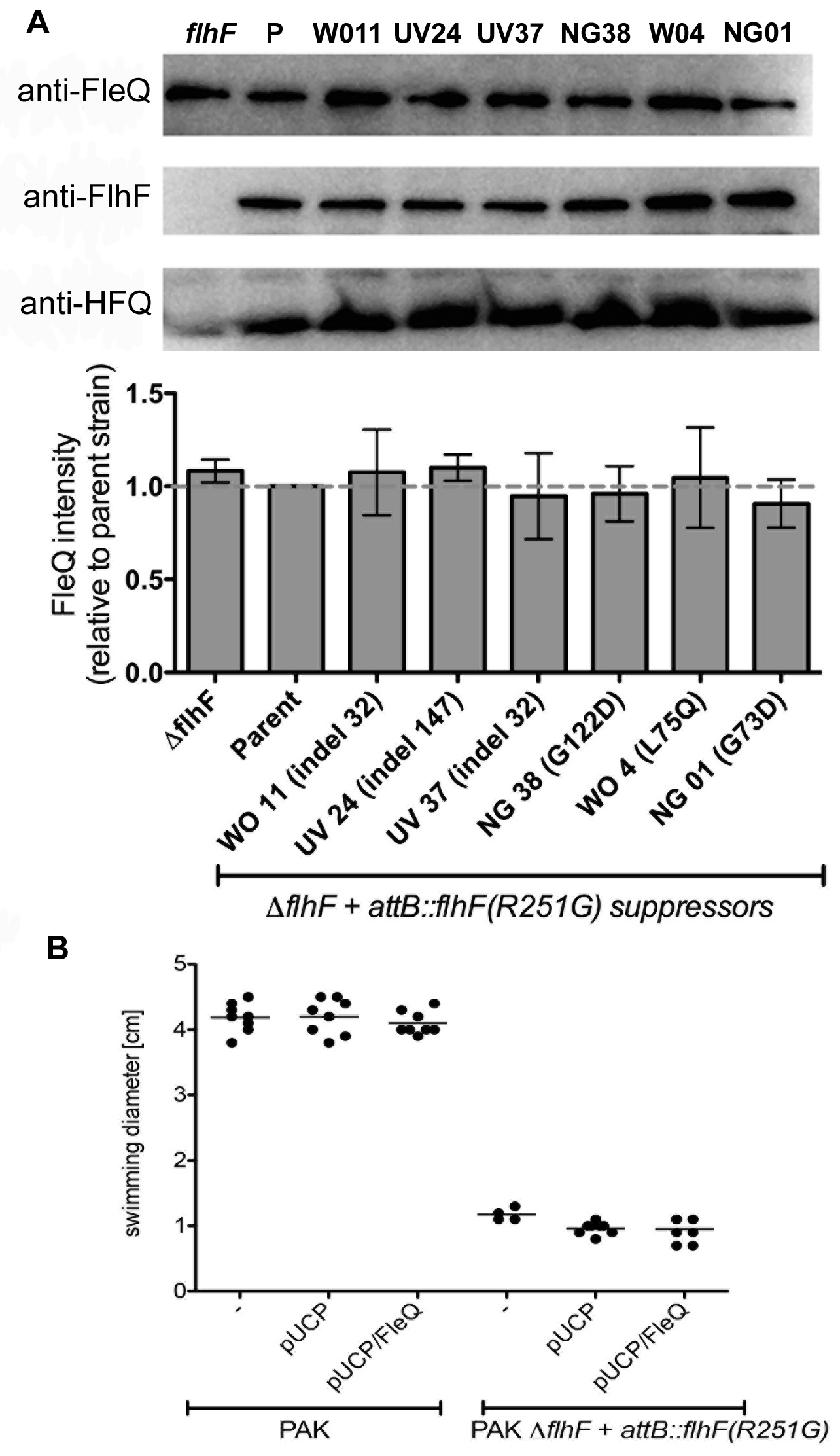

Supplement: S7 Fig — (A) Lysates prepared from overnight cultures grown in LB + 2% arabinose (ca. 1 x 10^7^ cells/lane) were separated by SDS-PAGE, transferred to PVDF and probed with antisera against FleQ, FlhF and Hfq (loading control). Chemiluminescence was used to detect and quantify antibody binding; the graph shows mean intensity ± SD for 3–6 replicates relative to the parent strain (parent (“P”), ΔflhF + attB::FlhF(R251G)). (B) Swimming motility was assayed on semisolid agar for wild-type and FlhF(R251G) overexpressing bacteria transformed with a FleQ expression construct (pUCP/FleQ) or vector control (pUCP). Each symbol indicates a replicate; the line indicates the mean. (TIF) [file ppat.1008149.s007.tif]

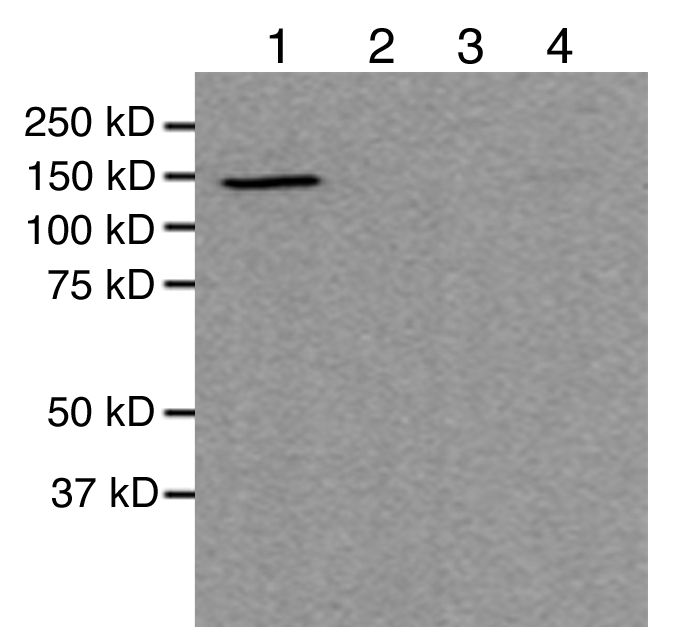

Supplement: S8 Fig — The BB2 epitope tag was recombined at the carboxy-terminal end of the endogenous fimV gene or the fimV(L7P) allele as described in Methods. Whole cell lysates corresponding to 2 x 10^8^ cells (except for lane 4, 6 x 10^8^ cells) were separated by SDS-PAGE, transferred to PVDF, and probed with anti-BB2 monoclonal antibody. Lane 1: PAK fimV-BB2, Lane 2: PAK, Lane 3: empty, Lane 4: PAK fimV(L7P)-BB2. (TIF) [file ppat.1008149.s008.tif]

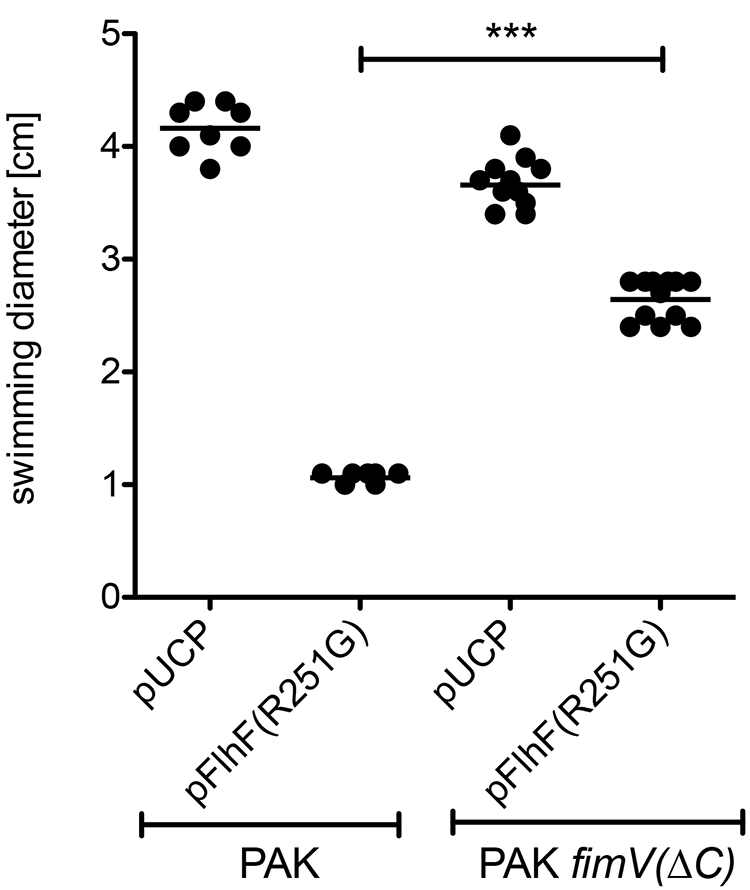

Supplement: S9 Fig — The carboxy-terminal domain of the chromosomal fimV gene was deleted, resulting in PAK fimV(ΔC). Overexpression of FlhF(R251G) from a plasmid inhibits swimming of PAK, but the dominant negative phenotype is significantly suppressed in the fimV(ΔC) background. Lines indicate the mean of 8–12 independent replicates. (***, p > 0.001; two-way ANOVA with Bonferroni posttest). (TIF) [file ppat.1008149.s009.tif]

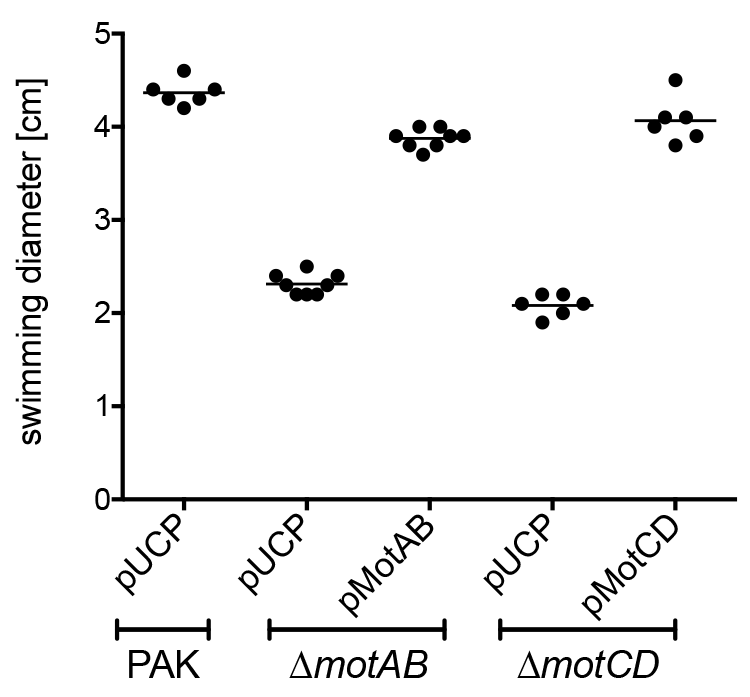

Supplement: S10 Fig — motAB and motCD genes were deleted by homologous recombination, and swimming behavior of the resulting mutant strains was assayed on 0.3% LB agar. Swimming diameters were measured for mutants (carrying empty pUCP vector) and for complemented strains as indicated (n = 6–8). Line indicates mean. (TIF) [file ppat.1008149.s010.tif]

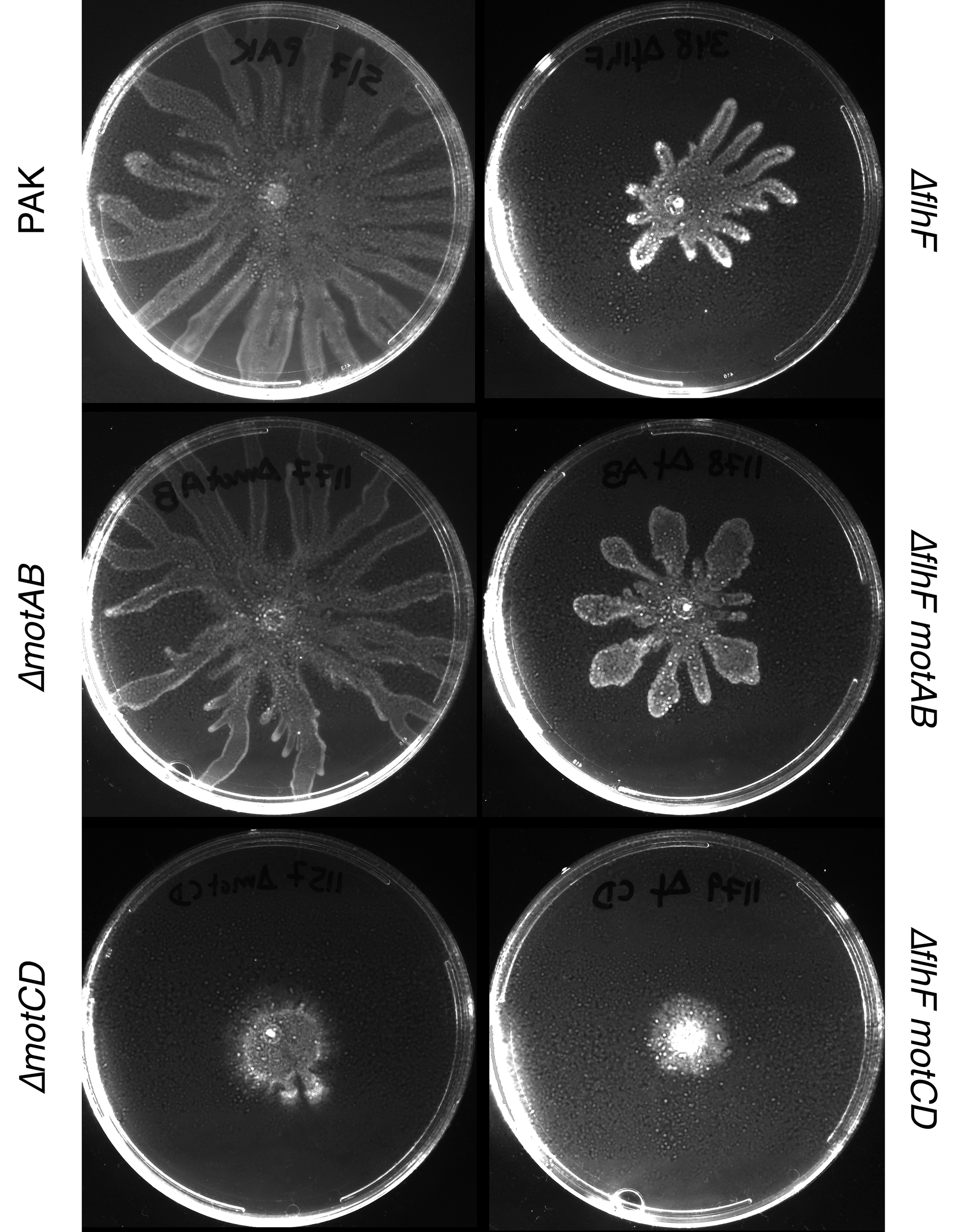

Supplement: S11 Fig — Unmarked deletions of the motAB and motCD genes were constructed by homologous recombination in PAK and ΔflhF backgrounds. Swarming was assayed on 0.5% agar as described in Methods. (TIF) [file ppat.1008149.s011.tif]

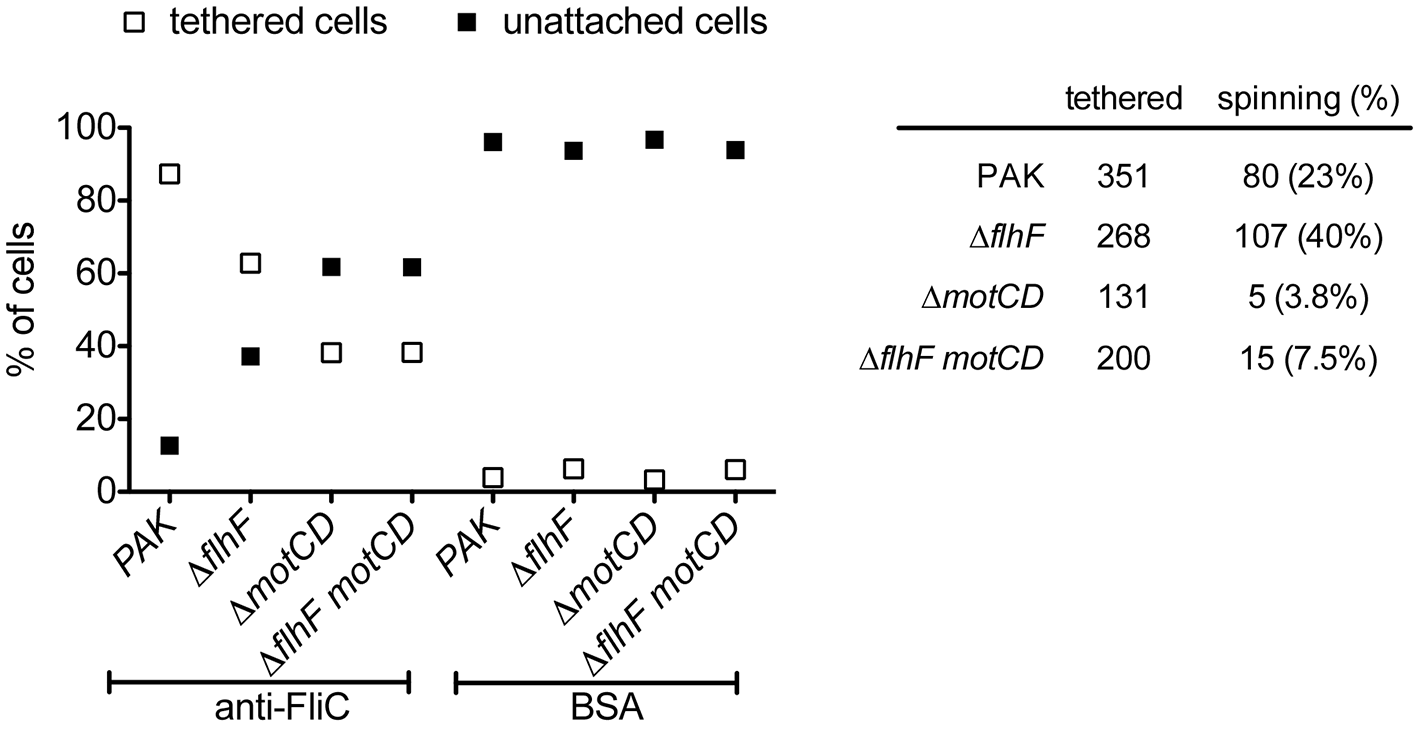

Supplement: S12 Fig — Tethered cells were imaged by videomicroscopy 5 minutes after incubation on anti-flagellin antibody coated or BSA coated slides. Percentage of unattached (open symbol) vs. tethered (solid symbol) cells are shown for PAK, ΔflhF, ΔmotCD, and ΔflhF motCD (n = 300–500 cells per strain/condition). The inset table shows the number of tethered vs. spinning cells for each strain with anti-flagellin coated slides. (TIF) [file ppat.1008149.s012.tif]
