# Supplementary material for: Modulation of flagellar rotation in surface-attached bacteria: A pathway for rapid surface-sensing after flagellar attachment
Source: PLoS Pathog. 2019 Nov 4;15(11):e1008149. doi: 10.1371/journal.ppat.1008149 (PMC6855561; doi:10.1371/journal.ppat.1008149)
Supplement: S2 Table — (DOCX) [file ppat.1008149.s014.docx]

**S2 Table. Bacterial strains and plasmids used in this study**

| **Strain or plasmid** | **Description or relevant genotype** | **Source or reference** |
| --- | --- | --- |
| *E. coli* strains |  |  |
| DH5α | Used for subcloning | Invitrogen |
| XL1-Blue | Used for subcloning | Stratagene |
| S17-1 | Used for mating constructs into *P. aeruginosa* | [1] |
| KDZif1∆Z | Used for bacterial two-hybrid assay | [2] |
| *P. aeruginosa* strains |  |  |
| PA14 | Wild type isolate | F. Ausubel |
| PAK | Wild type isolate | J. Mattick |
| PAK *∆fliC* | Deletion of *fliC (PA1092)* | J. Mattick |
| PAK *∆pilA* | Deletion of *pilA (PA4525)* | [3] |
| PAK *∆flhF* | In-frame deletion of amino acids 7-430 of FlhF *(PA1453)* | [4] |
| PAK *∆flhF pilA* | Deletion of *flhF* and *pilA* | This work |
| PAK *∆motAB* | Deletion of *motA (PA4954)* and *motB (PA4953)* | This work |
| PAK *∆motCD* | Deletion of *motC (PA1460)* and *motD (PA1461)* | This work |
| PAK *∆flhF ∆motAB* | Deletion of *motA (PA4954)* and *motB (PA4953)* in *∆flhF* | This work |
| PAK *∆flhF ∆motCD* | Deletion of *motC (PA1460)* and *motD (PA1461)* in *∆flhF* | This work |
| PAK *∆cheR1* | Deletion of *cheR1 (PA3348)* | This work |
| PAK *∆cheZ* | Deletion of *cheZ (PA1457)* | This work |
| PAK *∆fleN* | Deletion of *fleN (PA1454)* | This work |
| PAK ∆*fleN* *fimV(L7P)* | Double mutant | This work |
| PAK ∆*fleN* ∆*flhA* | Double mutant | This work |
| PAK *attB::flhF* | *FlhF-His6* under the control of an arabinose promoter integrated at the *attB* site (*attB*::p_ARA_ *flhF-His6)* | This work |
| PAK *attB::flhF(R251G)* | *FlhF(R251G)-His6* under the control of an arabinose promoter integrated at the *attB* site (*attB*::p_ARA_ *flhF (R251G)-His6)* | This work |
| PAK *∆flhF attB::flhF(R251G)* | *FlhF(R251G)-His6* under the control of an arabinose promoter integrated at the *attB* site in *∆flhF* background  (*attB*::p_ARA_ *flhF (R251G)-His6)* | [3] |
| NG01 | Suppressor of PAK *∆flhF* + *attB::flhF(R251G)* with *vfr(G73D)* | This work |
| PA14 *fimV::Tn* | Transposon insertion disrupting *fimV* | G. O’Toole |
| PA14 *pilI::Tn* | Transposon insertion disrupting *pilI* | G. O’Toole |
| PA14 *pilJ::Tn* | Transposon insertion disrupting *pilJ* | G. O’Toole |
| PA14 *pilG::Tn* | Transposon insertion disrupting *pilG* | G. O’Toole |
| PA14 *pilQ::Tn* | Transposon insertion disrupting *pilQ* | G. O’Toole |
| PAK *fimV(L7P)* | *fimV(L7P)* under its native promoter at the *fimV* site | This work |
| PAK *fimV(ΔC)* | *fimV(1-769)* under its native promoter at the *fimV* site | This work |
| PAK *fimV-BB2* | *fimV-BB2* under its native promoter at the *fimV* site | This work |
| PAK *fimV(L7P)-BB2* | *fimV(L7P)-BB2* under its native promoter at the *fimV* site | This work |
| PAK | Wild type isolate provided by M. Wolfgang; isogenic with adenylate cyclase mutants | [5] |
| PAK ∆*cyaA* | Deletion of cyaA | [5] |
| PAK ∆*cyaB* | Deletion of cyaB | [5] |
| PAK ∆*cyaAB* | Deletion of cyaA and cyaB | [5] |
| PAK *fliC(T394C)* | *fliC(T394C)* allele replacing chromosomal *fliC* | [6] |
| PAK ∆*fleN fliC(T394C)* | *fliC(T394C)* allele replacing chromosomal *fliC* | [6] |
| Plasmids |  |  |
| pUCP | Expression vector; Ap^R^ | [7] |
| pFlhF(R251G) | *flhF(R251G)* cloned in pUCP under *lac* promoter; Ap^R^ | This work |
| pMotAB | *motA* and *motB* cloned in pUCP under *lac* promoter; Ap^R^ | This work |
| pMotCD | *motC* and *motD* cloned in pUCP under *lac* promoter; Ap^R^ | This work |
| pFleQ | *fleQ* (*PA1097*) cloned in pUCP under *lac* promoter; Ap^R^ | [8] |
| pDONRX | Gateway-adapted suicide vector; Gm^R^ | [9] |
| pDONRX/*fimV-BB2* | Shuttle vector to introduce *fimV-BB2* into chromosome; Gm^R^ | This work |
| pDONRX/*fimV(L7P)* | Shuttle vector to introduce *fimV(L7P)* into chromosome; Gm^R^ | This work |
| pDONRX/*fimV(ΔC)* | Shuttle vector to introduce *fimV(1-769)* into chromosome; Gm^R^ | This work |
| pDONRX*/ΔmotAB* | Shuttle vector to delete *motA (PA4954)* and *motB (PA4953)*; Gm^R^ | This work |
| pDONRX/*ΔmotCD* | Shuttle vector to delete *motC (PA1460)* and *motD (PA1461)*; Gm^R^ | This work |
| pDONRX/*ΔcheR1* | Shuttle vector to delete *cheR1 (PA3348)*; Gm^R^ | This work |
| pDONRX/*ΔcheZ* | Shuttle vector to delete *cheZ (PA1457)*; Gm^R^ | This work |
| pDONRX/*ΔfleN* | Shuttle vector to delete *cbpA (PA1454)*; Gm^R^ | This work |
| pEX18Gm/*ΔpilA* | Shuttle vector to delete *pilA* (*PA4525*) ; Gm^R^ Tc^R^ | [10] |
| pMMB67EH | Shuttle vector; Ap^R^ | [11] |
| pMMB67EH/Vfr | Shuttle vector to express Vfr; Ap^R^ | [5] |
| pBRGPω | Vector for bacterial two hybrid assay with a C-TD reporter fusion of ω subunit of *E. coli* RNA polymerase; Ap^R^ | [2] |
| pACTR-AP-Zif | Vector for bacterial two hybrid assay with a C-TD reporter fusion of zinc finger DNA-binding domain of Zif; Tc^R^ | [2] |
| pBRGP / *flhF(WT)-ω* | Vector for bacterial two hybrid with FlhF(WT)-ω fusion; Ap^R^ | [3] |
| pBRGP / *flhF(R251G)-ω* | Vector for bacterial two hybrid with FlhF(R251G)-ω fusion; Ap^R^ | [3] |
| pBRGP / *flhF(K222A)-ω* | Vector for bacterial two hybrid with FlhF(K222A)-ω fusion; Ap^R^ | [3] |
| pBRGP / *flhF(D294A)-ω* | Vector for bacterial two hybrid with FlhF(D294A)-ω fusion; Ap^R^ | [3] |
| pACTR / *flhF(WT)*-Zif | Vector for bacterial two hybrid with FlhF(WT)-Zif fusion; Tc^R^ | [3] |
| pACTR / *flhF(R251G)*-Zif | Vector for bacterial two hybrid with FlhF(R251G)-Zif fusion; Tc^R^ | [3] |
| pACTR / *flhF(K222A)*-Zif | Vector for bacterial two hybrid with FlhF(K222A)-Zif fusion; Tc^R^ | [3] |
| pACTR / *flhF(D294A)*-Zif | Vector for bacterial two hybrid with FlhF(D294A)-Zif fusion; Tc^R^ | [3] |
| pBRGP / *flhF(L298R,P299L)-ω* | Vector for bacterial two hybrid with FlhF(L298R,P299L)-ω fusion; Ap^R^ | This work |
| pACTR / *flhF(*L298R,P299L)-Zif | Vector for bacterial two hybrid with FlhF(L298R,P299L)-Zif fusion; Tc^R^ | This work |
| pBRGP / *fimV(cyto)-ω* | Vector for bacterial two hybrid with FimV(aa 491-919)-ω fusion; Ap^R^ | This work |
| pBRGP / *fimV(cytoΔC)-ω* | Vector for bacterial two hybrid with FimV(aa 491-769)-ω fusion (aa 491-769); Ap^R^ | This work |
| pACTR / *fimV(cyto)*-Zif | Vector for bacterial two hybrid with FimV(aa 491-919)-Zif fusion; Tc^R^ | This work |
| pACTR / *fimV(cytoΔC)*-Zif | Vector for bacterial two hybrid with FimV(aa 491-769)-Zif fusion (aa 491-769); Tc^R^ | This work |
| pBRGP / *vfr-ω* | Vector for bacterial two hybrid with Vfr-ω fusion; Ap^R^ | This work |
| pACTR / *vfr*-Zif | Vector for bacterial two hybrid with Vfr-Zif fusion; Tc^R^ | This work |
| pBRGP / *fliF-ω* | Vector for bacterial two hybrid with FliF-ω fusion; Ap^R^ | This work |
| pACTR / *fliF*-Zif | Vector for bacterial two hybrid with FliF-Zif fusion; Tc^R^ | This work |
| pBRGP / *fliG-ω* | Vector for bacterial two hybrid with FliG-ω fusion; Ap^R^ | This work |
| pACTR / *fliG*-Zif | Vector for bacterial two hybrid with FliG-Zif fusion; Tc^R^ | This work |
| pBRGP / *FliM-ω* | Vector for bacterial two hybrid with FliM-ω fusion; Ap^R^ | This work |
| pACTR / *FliM*-Zif | Vector for bacterial two hybrid with FliM-Zif fusion; Tc^R^ | This work |
| pBRGP / *FliN-ω* | Vector for bacterial two hybrid with FliN-ω fusion; Ap^R^ | This work |
| pACTR / *FliN*-Zif | Vector for bacterial two hybrid with FliN-Zif fusion; Tc^R^ | This work |
| pBRGP / *motA-ω* | Vector for bacterial two hybrid with MotA-ω fusion; Ap^R^ | This work |
| pACTR / *motA*-Zif | Vector for bacterial two hybrid with MotA-Zif fusion; Tc^R^ | This work |
| pBRGP / *motB-ω* | Vector for bacterial two hybrid with MotB-ω fusion; Ap^R^ | This work |
| pACTR / *motB*-Zif | Vector for bacterial two hybrid with MotB-Zif fusion; Tc^R^ | This work |
| pBRGP / *motC-ω* | Vector for bacterial two hybrid with MotC-ω fusion; Ap^R^ | This work |
| pACTR / *motC*-Zif | Vector for bacterial two hybrid with MotC-Zif fusion; Tc^R^ | This work |
| pBRGP / *motD-ω* | Vector for bacterial two hybrid with MotD-ω fusion; Ap^R^ | This work |
| pACTR / *motD*-Zif | Vector for bacterial two hybrid with MotD-Zif fusion; Tc^R^ | This work |

References

1. Simon R, Priefer U, Puhler A. A broad host range mobilization system for in vivo genetic engineering: transposon mutagenesis in Gram negative bacteria. Biotechnology. 1983;1:784-91.

2. Vallet-Gely I, Donovan KE, Fang R, Joung JK, Dove SL. Repression of phase-variable cup gene expression by H-NS-like proteins in *Pseudomonas aeruginosa*. Proc Natl Acad Sci U S A. 2005;102(31):11082-7. Epub 2005/07/27. doi: 10.1073/pnas.0502663102. PubMed PMID: 16043713; PubMed Central PMCID: PMC1182424.

3. Schniederberend M, Abdurachim K, Murray TS, Kazmierczak BI. The GTPase Activity of FlhF Is Dispensable for Flagellar Localization, but Not Motility, in *Pseudomonas aeruginosa*. J Bacteriol. 2013;195(5):1051-60. Epub 2012/12/25. doi: 10.1128/JB.02013-12. PubMed PMID: 23264582.

4. Murray TS, Kazmierczak BI. FlhF is required for swimming and swarming in *Pseudomonas aeruginosa*. J Bacteriol. 2006;188(19):6995-7004.

5. Wolfgang MC, Lee VT, Gilmore ME, Lory S. Coordinate regulation of bacterial genes by a novel adenylate cyclase signaling pathway. Devlop Cell. 2003;4:253-63.

6. Zhu S, Schniederberend M, Zhitnitsky D, Jain R, Galan JE, Kazmiercak BI, et al. *In situ* structures of polar and laterla flagella revealed by cryo-electron tomography. J Bacteriol. 2019;201(13):e00117-19.

7. Watson AA, Alm RA, Mattick JS. Construction of improved vectors for protein production in *Pseudomonas aeruginosa*. Gene. 1996;172:163-4.

8. Jain R, Kazmierczak BI. A Conservative Amino Acid Mutation in the Master Regulator FleQ Renders *Pseudomonas aeruginosa* Aflagellate. PLoS One. 2014;9(5):e97439. Epub 2014/05/16. doi: 10.1371/journal.pone.0097439. PubMed PMID: 24827992.

9. Fulcher NB, Holliday PM, Klem E, Cann MJ, Wolfgang MC. The *Pseudomonas aeruginosa* Chp chemosensory system regulates intracellular cAMP levels by modulating adenylate cyclase activity. Mol Microbiol. 2010;76(4):889-904. Epub 2010/03/30. doi: 10.1111/j.1365-2958.2010.07135.x. PubMed PMID: 20345659; PubMed Central PMCID: PMC2906755.

10. de Kerchove AJ, Elimelech M. Impact of alginate conditioning film on deposition kinetics of motile and nonmotile *Pseudomonas* *aeruginosa* strains. Appl Environ Microbiol. 2007;73:5227-34.

11. Furste JP, Pansegrau W, Frank R, Blocker H, Scholz P, Bagdasarian M, et al. Molecular cloning of the plasmid RP4 primase region in a multi-host range *tacP* expression vector. Gene. 1986;48:119-31.
